# Soil flooding filters evolutionary lineages of tree communities in Amazonian riparian forests

**DOI:** 10.1002/ece3.11635

**Published:** 2024-07-23

**Authors:** Sthefanie do Nascimento Gomes de Souza, Darlisson Mesquita Batista, Adriano Costa Quaresma, Ana Luiza Costa, Layon Oreste Demarchi, Bianca Weiss Albuquerque, Viviane Pagnussat Klein, Gildo Feitoza, Angélica Faria de Resende, Gisele Biem Mori, Florian Wittmann, Leidiane Leão Oliveira, Amanda Frederico Mortati, Alan Cavalcanti da Cunha, Jochen Schongart, Aline Lopes, Maria Teresa Fernandez Piedade, Thiago André

**Affiliations:** ^1^ Postgraduate Program in Ecology National Institute for Amazon Research (INPA) Manaus Brazil; ^2^ Ecology, Monitoring and Sustainable Use of Wetlands (MAUA Research Group) National Institute for Amazon Research (INPA) Manaus Brazil; ^3^ Postgraduate Program in Biodiversity Federal University of Western Pará Santarém Brazil; ^4^ Institute of Technology (KIT) Karlsruhe Germany; ^5^ Forest Sciences Department, ESALQ/USP University of São Paulo São Paulo Brazil; ^6^ Institute of Water Sciences and Technology Federal University of Western Pará Santarém Brazil; ^7^ Center for Sustainable Development University of Brasília Brasília Brazil; ^8^ Program in Environmental Science (PPGCA), Federal University of Amapá (UNIFAP) Macapá Brazil; ^9^ Researcher at the Cesumar Institute of Science, Technology and Innovation (ICETI) Maringá Brazil; ^10^ Department of Botany, Institute of Biological Sciences University of Brasília Brasília Brazil

**Keywords:** endemism, overdispersion, phylogenetic diversity, randomness, wetlands

## Abstract

Inundations in Amazonian black‐water river floodplain result in the selection of different tree lineages, thus promoting coexistence between species. We investigated whether Amazonian tree communities are phylogenetically structured and distributed along a flooding gradient from irregularly flooded forests along streams embedded within upland (*terra‐firme*) forest to seasonally flooded floodplains of large rivers (*igapós*). Floristic inventories and hydrological monitoring were performed along the Falsino River, a black‐water river in the eastern Amazon within the Amapá National Forest. We constructed a presence‐and‐absence matrix and generated a phylogeny using the vascular plant database available in GenBank. We calculated the standardized values of the metrics of phylogenetic diversity (ses.PD), average phylogenetic distance (ses.MPD), and average nearest‐neighbor distance (ses.MNTD) to test whether the history of relationships between species in the community is influenced by inundation. We used the phylogenetic endemism (PE) metric to verify the existence of taxa with restricted distribution. Linear regressions were used to test whether phylogenetic metrics have a significant relationship with the variables: maximum flood height, maximum water table depth, and maximum flood amplitude. The results show that forests subject to prolonged seasonal flooding have reduced taxon richness, low phylogenetic diversity, and random distribution of lineages within communities. On the other hand, *terra‐firme* riparian forests showed higher rates of taxon richness, diversity, and phylogenetic dispersion, in addition to greater phylogenetic endemism. These results indicate that seasonal and predictable soil flooding filters tree lineages along the hydrographic gradient. Different adaptations to root waterlogging are likely requirements for colonization in these environments and may represent an important factor in the diversification of tree lineages in the Amazon biome.

## Graphical abstract

Flooding in the floodplain of Amazonian black‐water rivers results in the selection of different tree lineages, thus promoting coexistence between species. We investigated whether Amazonian tree communities are phylogenetically structured and distributed along a flooding gradient, from irregularly flooded forests along streams embedded in highland forests (*terra‐firme*) to seasonally flooded floodplains of large rivers (*igapós*). The results show that forests subject to prolonged seasonal flooding had reduced taxon richness, low phylogenetic diversity, and random distribution of lineages within communities, while riparian forests in streams had higher rates of taxon richness, diversity, and phylogenetic dispersion, in addition to greater phylogenetic endemism. Different adaptations to root waterlogging are likely requirements for colonization in these environments and may represent an important factor in the diversification of tree lineages in the Amazon biome.

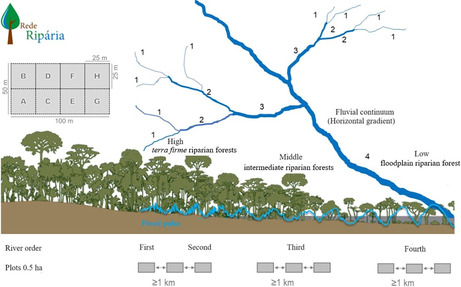

## 1 INTRODUCTION

The Amazon basin extends over 6.8 million km^2^ and presents a high richness and diversity of species (Antonelli & Sanmartín, 2011; Junk et al., 2011; Myers et al., 2000; ter Steege et al., 2013). It is a vast and interconnected system composed of different landscapes of upland (i.e., *terra‐firme*) forests and wetlands, linked by an extensive drainage system that has undergone significant changes throughout its history (Hoorn et al., 2010). This basin regulates a wide range of aquatic and terrestrial environments that are characterized by a diverse array of tree species adapted to different soil conditions and hydrological gradients (Junk et al., 1989, 2011; Prance, 1980; Salati & Vose, 1984). Here, we divide Amazonian forests into *terra‐firme* (upland) forests, which lie above the maximum flood level of rivers, and lowland floodplains that are either seasonally or permanently inundated by its marginal water body (Bredin et al., 2020; Sioli, 1985; Sombroek, 2000). The oligotrophic and sediment‐poor transparent or black water drain the oldest geological formations of the Amazon, predominantly formed during the Tertiary period, with alluvial sediments originating from Precambrian‐aged rocks due to the erosion of the Guiana and Central Brazilian Shields (Sioli, 1985; Irion et al., 1997). These types of flooded forests (*igapós*) exhibit nutrient scarcity and intense weathering, resulting in high endemism and low species richness compared to the *várzeas*, floodplain forests inundated by the more recent white‐water sediment‐rich rivers (Ayres, 1986; Fittkau, 1971; Junk et al., 1989; Kubitzki, 1987; Prance, 1979). Many tree species in *igapó* riparian forests are ecologically specialized to this ecosystem, leading to distinct floristic compositions when compared to *várzea* and *terra‐firme* riparian forests (Householder et al., 2024; Kubitzki, 1989), with a unique evolutionary signature of its phylogenetic composition (Luize et al., 2024).

Riparian zones form at the dynamic contacts between terrestrial and aquatic ecosystems, marginal to water bodies, showcasing diverse patterns in relation to the organization, diversity, and dynamics of amphibious communities (Naiman & Décamps, 1997). Riparian vegetation varies with soil water saturation, which increases from low‐order rivers in the catchment area toward high‐order rivers in the lowlands with simultaneously changing flood‐pulse patterns (Gregory et al., 1991; Junk et al., 1989, 2011). The flood pulse modifies the environment quickly in time and space (Junk et al., 1989). Seasonal flooding and deeper water tables in lowland regions, where flood pulses are regular and of greater amplitude (i.e., *igapó*), result in the predominance of species adapted to the long‐term flooding of several months (Koga et al., 2024; Parolin, 2009; Parolin & Wittmann, 2010; Piedade et al., 2013; Wittmann et al., 2013). In contrast, forests along streams and small rivers embedded within uplands (*terra‐firme* riparian forests), where the water table is shallow and closely related to surface water levels, experience stochastic, unpredictable, and low‐amplitude flood regimes. These environments harbor a higher diversity of species adapted to heterogeneous soil composition, facing different selective pressures from those found in large‐river floodplains. This results in a distinct species composition along the riparian zone of freshwater bodies (Junk et al., 2011; Kubitzki, 1989; Prance, 1980). Upland riparian environments generally feature poorly drained soils compared to those of large‐river floodplains (Drucker et al., 2008; Schietti et al., 2014), requiring adaptations from colonizing trees. The various types of tree species adaptations to the floodable areas of the Amazon basin are well documented (e.g., Da Silva et al., 2023; Ferreira et al., 2010; Junk et al., 2015; Parolin, 2009; Piedade et al., 2013).

Riparian forests along low‐order rivers bridge the gap between the *terra‐firme* and the alluvial plains of large rivers, with many tree species found in both floodable and upland areas. The concept of floodplain species colonization, proposed by Wittmann et al. (2010), suggests that the colonization of tree species in the alluvial plains of large rivers originated from the species pool of the *terra‐firme* over evolutionary periods, as the species gradually developed increasingly sophisticated adaptations to tolerate longer floods. However, further phylogenetic studies along the river continuum as suggested by Vannote et al. (1980) are necessary to provide additional support for the idea of species colonization in floodplains, highlighting the evolutionary processes shaping the diversity and distribution of Amazonian tree species across different habitats (Wittmann et al., 2022).

The complex interaction between hydrological factors that result in the flooding gradient plays a determining role in the organization of the different phytophysiognomies in the Amazon, considering the remarkable ability of plants to adapt to changing environmental conditions (Junk & Piedade, 1997; Parolin, 2009). Tree species of various taxonomic groups may exhibit convergent morphological and physiological adaptations, such as adventitious and aerenchyma roots, stem hypertrophy, lenticels, and reduced photosynthesis, in response to water stress (Ferreira et al., 2006; Koga et al., 2024; Kozlowski, 1997; Lobo & Joly, 1995; Wittmann & Parolin, 1999); whereas species composition and richness vary significantly both among and within floodable forest communities in the Amazon (Ferreira et al., 2005; Hoorn et al., 2017; Lobo et al., 2019; Rocha et al., 2019).

Despite its vastness and species richness, knowledge gaps exist regarding areas with high phylogenetic diversity (PD) (Webb, 2000) and phylogenetic endemism (PE) in Amazonian riparian zones (Rosauer et al., 2009). Phylogenetic diversity quantifies the evolutionary history of organisms based on ancestral relationships and is considered crucial for identifying areas with high diversity, while species endemism indicates the presence of taxa with restricted spatial distribution (Faith, 1992; Magurran, 2004; Owen et al., 2019; Ricotta, 2005; Vane‐Wright et al., 1991; Webb, 2000). Areas with high phylogenetic endemism play a vital role in biodiversity conservation (Ferrier et al., 2000; Pressey et al., 1994) and act as nature reserves for maximizing local phylogenetic diversity (Faith et al., 2004; Forest et al., 2007). These areas are extremely important in the preservation of species and in ensuring the maintenance of evolutionary diversity, considering the alarming loss of biodiversity at the global level (Cadotte et al., 2008; Dirzo & Raven, 2003; Isbell et al., 2023).

Factors, such as habitat characteristics and species traits, can influence patterns of phylogenetic richness and diversity (Cadotte et al., 2010; Ricklefs et al., 2014; Stropp et al., 2011). Environmental factors can function as filters, selecting species that are phylogenetically close or distant, and which may share similar functional characteristics to exploit the same resources in different ways (Fukami et al., 2005; Pausas & Verdú, 2010; Webb, 2000). This means that the coexistence of species can involve both those that are evolutionarily close (clustered) and those that are distant (overdispersed), or it can result in randomly distributed communities in space. Understanding these phylogenetic patterns is critical in order to obtain more accurate estimates of how plant communities are structured in space and time (Fine & Kembel, 2011; Gerhold et al., 2018; Kubitzki, 1987; Muscarella et al., 2020; Wittmann et al., 2010).

Recently, Luize et al. (2024) demonstrated that numerous tree lineages serve as significant indicators of geographic regions in Amazonia. The authors emphasized that *terra‐firme* and white‐water floodplain forests (*várzea*) exhibit the most variable phylogenetic composition, while white‐sand forests (*campinarana*) and black‐water floodplain forests (*igapó*) tend to have less variable phylogenetic composition. Here, we focus on a local river basin scale, where we evaluated two hypothetical scenarios to unravel historical evolutionary processes: (1) a scenario where only a few tree lineages have evolved traits that enable survival in floodable areas, resulting in a phylogenetically clustered community; or (2) a scenario where independent lineages of tree species have repeatedly colonized these environments, leading to convergences of evolutionary strategies for surviving flooding and thus resulting in reduced phylogenetic clustering along a gradient from upland small‐streams riparian forests (*terra‐firme*) to large‐river floodplain forests (*igapós*). Therefore, the main objective of this study was to analyze the diversity and phylogenetic structure of tree communities, ranging from riparian forests adjacent to small streams on *terra‐firme*, i.e., upper lands, with brief, shallow, and unpredictable flooding, to large rivers on *igapó*, i.e., lower lands, with prolonged, deep, and predictable flooding. The analysis aimed to establish relationships between these environments and the hydrological gradients of flooding, above and below the soil surface, in order to enhance our understanding of the organization of tree communities in Amazonia, which thrive amidst a mosaic of these distinct conditions.

## 2 MATERIALS AND METHODS

### 2.1 Study site

**FIGURE 1:**
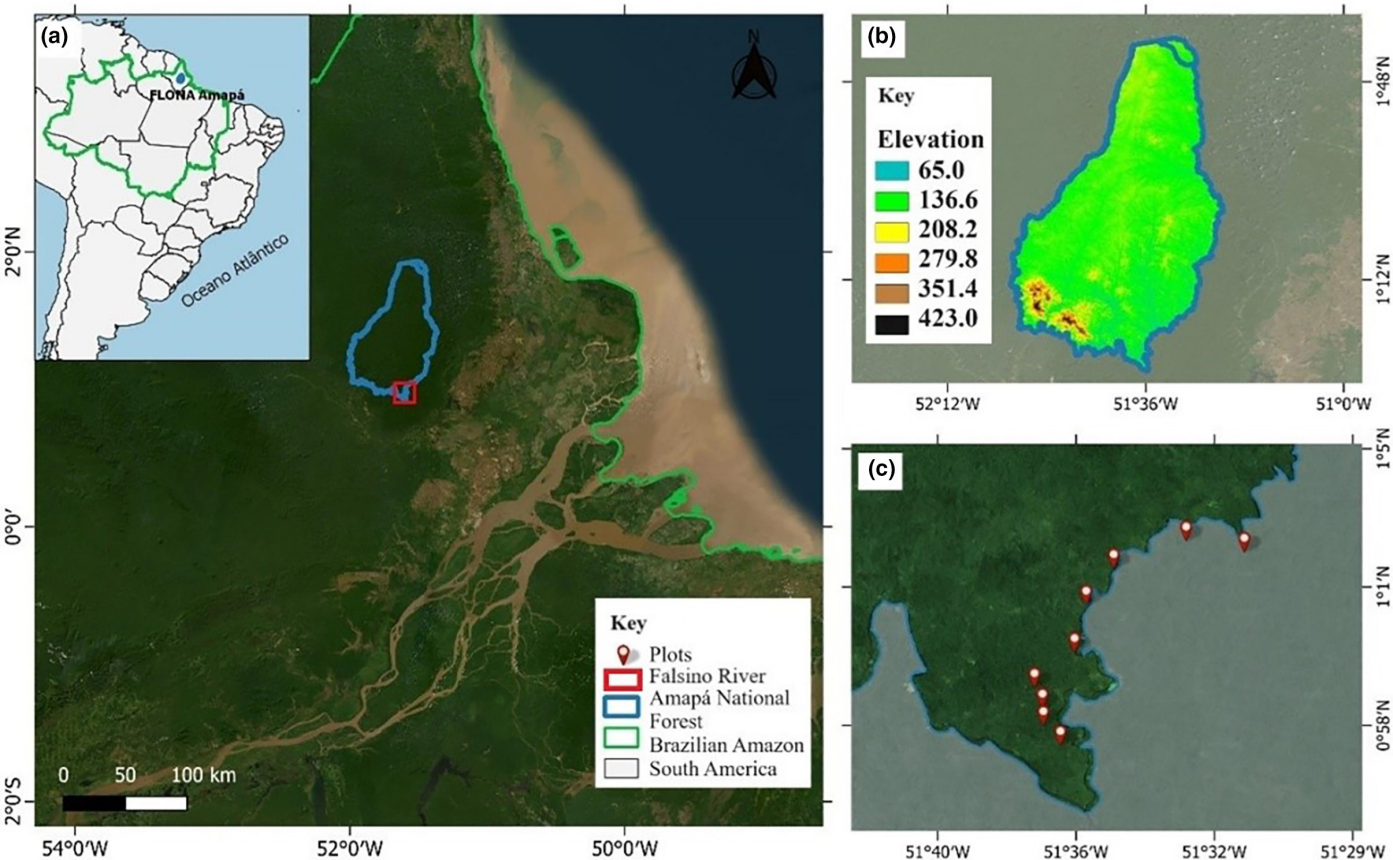
(a) Location of the study area in the Amapá National Forest; (b) Digital surface elevation model (MDE Copernicus); and (c) Location of the plots. *Source*: Geoprocessing and Modeling Center – MAUA /INPA Group.

This study was conducted in the oligotrophic black‐water sub‐basin of the Falsino River (0°55' N and 51°35' W), situated adjacent to the Amapá National Forest (FLONA Amapá), in the state of Amapá, Brazil (Figure 1). Covering an area of 460,353 ha, FLONA Amapá borders the Amapá State Forest to the east and the Montanhas do Tumucumaque National Park to the west, collectively forming the Amapá biodiversity corridor, which spans over 70% of the state's total area (ICMBIO, 2014). The perimeter of FLONA Amapá is delineated by the Araguari River to the south, the Falsino River to the east, and the Mutum River to the west. The Falsino River, a major tributary of the Araguari River, flows more than 70 km upstream from the Porto Grande hydroelectric dam, remaining in its natural state and accessible only with authorization for scientific research or tourism (ICMBIO, 2016). With its most distant headwaters in the Guiana Shield, this black‐water river carries a low sediment and nutrient load (Junk et al., 2015). Historically, the Araguari River discharged into the Atlantic Ocean; however, due to erosion of the Urucurituba channel, most of its water now diverts to the Amazon River (Da Silva et al., 2023; dos Santos et al., 2018).

The climate of the FLONA Amapá region is tropical hot‐humid type, with an average temperature of 27.6°C in Macapá and an average annual rainfall of 2,284 mm in the Serra do Navio region, the closest municipalities with available data (SUDAM, 1984). Situated in the Guiana Shield region, FLONA Amapá features geological formation dating from the Precambrian (>1,000 Mya) and Paleoproterozoic periods (2,300–2,050 Mya) (Gómez et al., 2019). The terrain ranges from 50 m above sea level (a.s.l.) to peaks reaching 460 m a.s.l. in the southern part of the protected area (ICMBIO, 2016). The vegetation is heterogeneous, encompassing *terra‐firme* forests, lowlands, flooded forests, and transitional zones between forests and savannas (ICMBIO, 2014).

### 2.2 Sampling design

**FIGURE 2:**
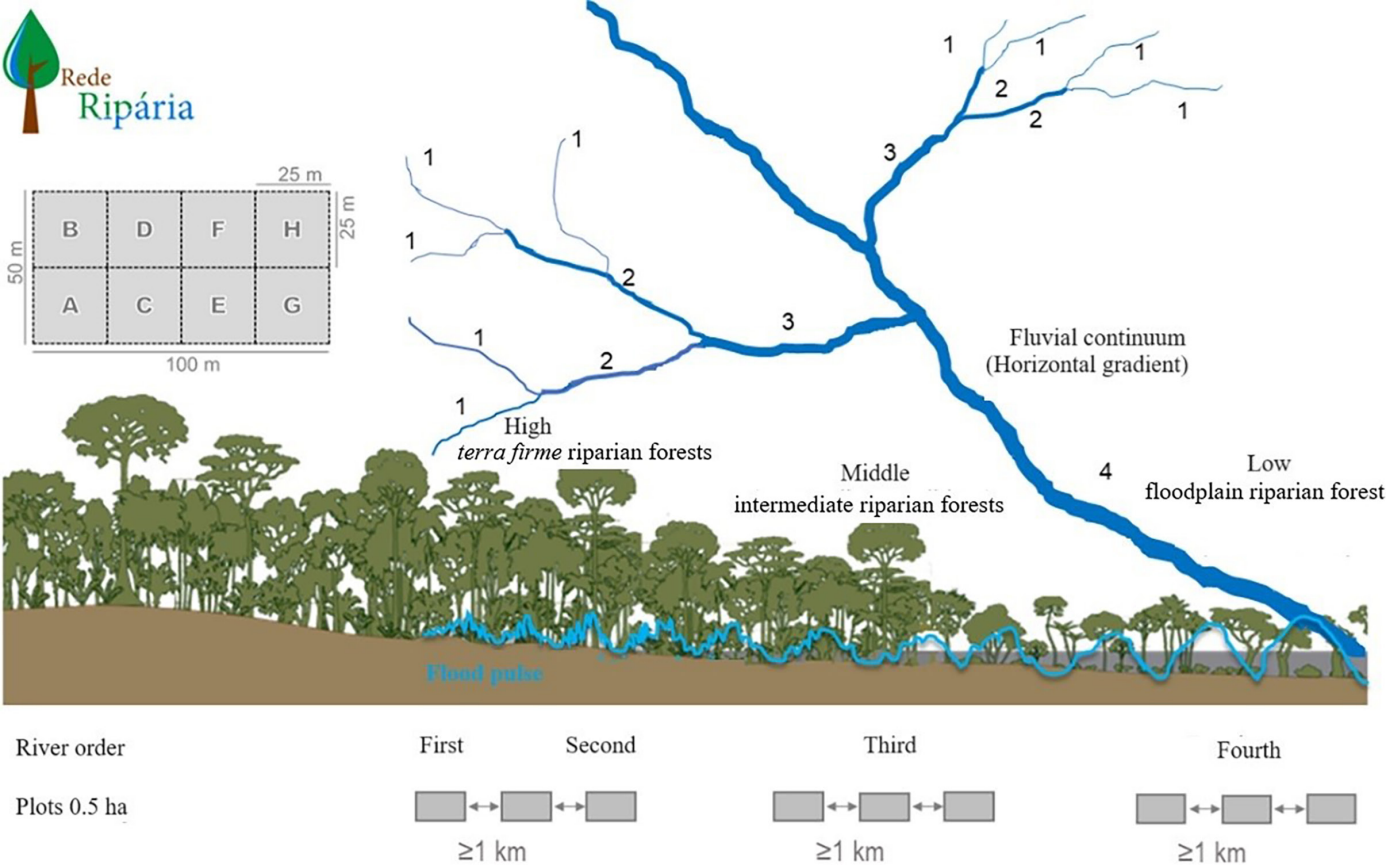
Sampling design of the distribution of plots of 0.5 ha (100 m × 50 m) subdivided into eight subplots of 625 m^2^ allocated along the river orders; The first‐ to second‐order rivers (shallows) are represented by riparian forests in the upper section, the third‐order rivers have intermediate conditions represented by the middle section, and the fourth‐order rivers have flooded forest formations (*igapós*) represented by the low section. The river continuum corresponds to all sampling carried out from the headwaters to the mouth of the sub‐basin. *Source*: Jochen Schöngart/Rede Ripária.

The floristic inventory included trees with a diameter at breast height (DBH) of ≥10 cm and was conducted in August 2018. Species identification in the field was carried out with the assistance of a botany taxonomist. Additionally, we collected botanical vouchers in the field and deposited them at the INPA (National Institute for Amazon Research) herbarium in Manaus‐AM‐Brazil for final identification. The sub‐basin survey comprised nine plots of 0.5 ha (100 m × 50 m), each of them subdivided into eight subplots of 625 m^2^ to minimize within‐plot variation in altitude, soil composition, and water table depth. The plots were distributed along the sub‐basin's different flood regimes as follows: three in first‐ or second‐order small streams in the upper stretches (*terra‐firme* riparian forests), three along third‐order rivers in the middle stretches (intermediate riparian forests), and three in the floodplains of the main fourth‐order river in the lower stretches (*igapó* riparian forest; Figure 2). Plot locations were selected based on topography, maintaining a spacing of over 1 km between them to maximize sampling independence. The standardization of the sampling units enabled capturing environmental variation along the fluvial continuum from the headwaters to the mouth of the Falsino River.

### 2.3 Hydrological gradient monitoring

To measure flood height above the soil surface, linimetric rulers were installed along the channel bank closest to the plot, affixed to healthy, resistant trees using nails. These aluminum rulers, 3 m in length, were graduated every 1 cm and numbered every 2 cm. Monthly water level monitoring was conducted over a 1‐year period (Belúcio, 2020). In cases where flooding exceeded 3 m, a spare ruler was used alongside the installed one for measurement. Water table depth was determined by collecting water fluctuation data from piezometric wells with depths of 3–6 m, present in each plot. Flood amplitude was estimated based on the difference between maximum flood height and maximum water table depth using hydrological data monitoring.

### 2.4 Species relationships and phylogenetic community structure

The V.PhyloMaker package (Jin & Qian, 2019) was employed to reconstruct the phylogenetic hypothesis of tree species based on the presence‐and‐absence matrix of species inventoried. V. PhyloMaker utilizes an updated and expanded version of the GBOTB (GenBank taxa with a backbone provided by Only Tree of Life version 9.1) mega‐phylogeny developed by Smith and Brown (2018). This mega‐phylogeny encompasses all existing seed plant families (74,533 species) according to the APG IV system (Angiosperm Phylogeny Group 2016), rendering it the most extensive dated phylogeny for vascular plants available (Jin & Qian, 2019). Species absent in the GBOTB mega‐phylogeny but present in our dataset were added to their respective genera using scenario 3 with the scenarios = “S3” function implemented in V.PhyloMaker. The resulting phylogeny includes 33,3% of unresolved taxa (polytomies).

Species richness was calculated as the number of species in each plot, and raw values of phylogenetic diversity (PD) (Faith, 1992), mean phylogenetic distance (MPD), and mean nearest taxon distance (MNTD) were determined. To address a possible bias related to these three phylogenetic metrics and raw values of PD, MPD, and MNTD is their sensitivity to species richness present in the community (Cadotte et al., 2010; Sandel, 2018; Tucker & Cadotte, 2013). We removed the richness effect from the raw metric values (PD, MPD, and MNTD) (Kembel et al., 2010; Miller et al., 2017; Webb et al., 2002) and used the null model of the standardized effect size (ses.PD, ses.MPD, and ses.MNTD). The phylogenetic structure analyses were conducted using the ‘picante’ package, version 1.8.2 (Kembel et al., 2010). The null model of standardized effect size (ses.PD, ses.MPD, and ses.MNTD) was applied. The null model involved performing 999 permutations using the “independent swap” method, implemented in the R ‘picante’ package, following manual instructions. This method randomly rearranges the labels of the taxa in the phylogenetic distance matrix and allows for the calculation of the mean and standard deviation (SD) of the values obtained from the models generated by these permutations (Kembel et al., 2010). The “independent swap” model assumes that the ability of a species to colonize a plot is proportional to its frequency in the database, with each species having an equal probability of occurring in each plot (Gotelli & Graves, 1996; Hardy, 2008). Positive values of ses.PD suggest greater diversity, ses.MPD and ses.MNTD with *p* ≥ .95 indicate phylogenetic overdispersion, negative values indicate lower diversity, *p* ≤ .05 indicate phylogenetic clustering, and values around zero and without significance (*p* between .05 and .95) indicate a random phylogenetic pattern.

In addition to standardized ses.PD, ses.MPD, and ses.MNTD metrics, we also used the phylogenetic endemism (PE) metric that calculates the fraction of branches restricted to specific regions. For this analysis, we used the phyloregion package in the R program via the function “results_PE<‐phylo_endemism.” PE identifies areas or communities that harbor restricted components of phylogenetic diversity and is a metric to aid conservation studies by establishing criteria to prioritize regions to be conserved based on evolutionary importance (parts of the phylogeny with limited spatial distribution) (Rosauer et al., 2009). The phylogenetic endemism (PE) index considers all the communities as the maximum spatial range. For example, if all species occur in all communities, the PE value would be 1, indicating low phylogenetic endemism.

### 2.5 Statistical analysis

Phylogenetic structure analyses were performed using the ‘picante’ package, version 1.8.2 (Kembel et al., 2010). We used the “lm ()” function to conduct simple linear regressions and tested whether the three standardized phylogenetic metrics (ses.PD, ses.MPD, and ses.MNTD) were significantly related to flood amplitude, maximum flood height, and maximum water table depth. All analyses were performed in the program R, version 4.1.1 (R Core Team, 2023).

## 3 RESULTS

### 3.1 Tree inventory

The study included a total of 2,432 individuals, comprising 218 species, 133 genera, and 44 families. The phylogeny of all species is presented in the Data S1. Of the 2,432 individuals identified, 825 were situated in *terra‐firme* riparian forests, 869 in intermediate positions, and 738 in *igapó* riparian forests. Among the 44 families recorded, the five most species‐rich families were Fabaceae, Lecythidaceae, Chrysobalanaceae, Sapotaceae, and Myrtaceae. Noteworthy among the most species‐rich genera were *Eschweilera*, *Inga*, *Protium*, *Pouteria*, *Eugenia*, and *Buchenavia* (a list of species inventoried in the Falsino River is presented in Data S1).

Among the 10 most abundant species were *Euterpe oleracea* Mart., *Macrolobium bifolium* (Aubl.) Pers., *Myrcia umbraticola* (Kunth) E. Lucas & C.E. Wilson, *Licania micrantha* Miq., *Pterocarpus santalinoides* L'Hér. ex DC., *Cynometra hostmanniana* Tul., *Hymenopus macrophyllus* (Benth.) Sothers & Prance, *Discocarpus essequeboensis* Klotzsch, *Lecythis idatimon* Aubl., and *Leptobalanus sprucei* (Hook.f.) Sothers & Prance. Of the 218 species identified, 75 were represented by only one individual, while 29 species were represented by two individuals.

When examining family distribution along the hydrographic gradient, we observed that *terra‐firme* forests presented a higher number of taxa with restricted spatial distribution, indicating the absence of close relatives in the community. The families Anacardiaceae, Arecaceae, Bignoniaceae, Goupiaceae, Lamiaceae, Lauraceae, Linaceae, Nyctaginaceae, Olacaceae, Stemonuraceae, and Urticaceae were exclusively present in these forests. Similarly, the families Polygonaceae and Putranjivaceae occurred exclusively in flooded forests.

### 3.2 Hydrological gradient

**FIGURE 3:**
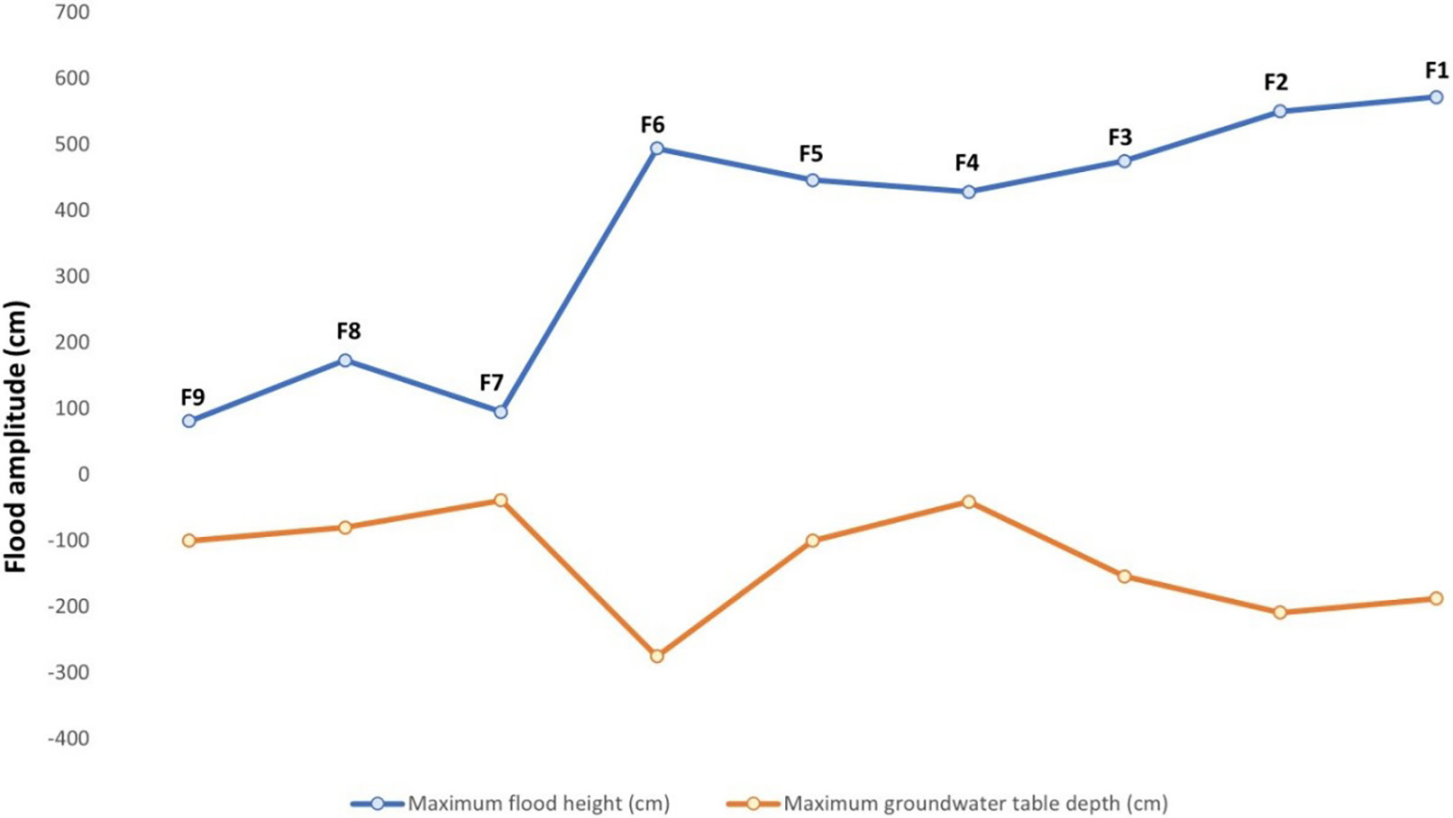
Mean of the annual variation of the hydrological gradient in the plots sampled in different stretches in the Amapá National Forest.

In *terra‐firme* small‐streams riparian forests, the average flooding height was 116.33 cm, with a standard deviation (SD) of 44.11 cm. The mean of the maximum depth of the water table was −73 cm (±40.42 cm). The average amplitude was approximately 189.33 cm (±49.17 cm). In the riparian forests from the intermediate zone, the average flooding height was 456 cm (±29.98), while the mean of the maximum depth of the water table was −138.67 cm (±100.16 cm). The average amplitude was 594.67 cm (±133.66 cm). In the *igapó* riparian forests, the average flooding height was 532.33 cm (±46.39 cm), while the mean of the maximum depth of the water table was −183.5 cm (±22.56 cm). The average amplitude was 715.17 cm (±64.76 cm; Figure 3 and Table 1).

**TABLE 1:** Standardized effect size of phylogenetic diversity metrics (ses.PD), mean phylogenetic distance (ses.MPD), and the mean phylogenetic distance between the closest taxa (ses.MNTD), phylogenetic endemism (PE) index, and environmental variables observed in the plots of the Falsino River.

Plot	ses.PD	*p*	ses.MPD	*p*	ses.MNTD	*p*	PE	Alt	Mwt	Ampl
*terra‐firme* riparian forests	1.58	**.95**	1.96	.84	1.83	**.96**	19.09	81	−100	181
*terra‐firme* riparian forests	−1.02	.13	−0.38	.22	−0.79	.28	9.11	173	−80	253
*terra‐firme* riparian forests	−1.28	.10	−1.29	.14	−1.05	.15	4.58	95	−39	134
Intermediate riparian forests	0.97	.23	−0.03	.24	−0.73	.25	5.21	494	−275	769
Intermediate riparian forests	−0.95	.20	−0.68	.44	−0.16	.47	4.09	446	−100	546
Intermediate riparian forests	−0.91	.25	−0.20	.44	−0.17	.46	4.25	428	−41	469
Foodplain riparian forests	0.60	.56	−0.73	.79	0.79	.80	5.89	475	−154	629
Foodplain riparian forests	0.99	.84	0.58	.74	0.68	.76	6.37	550	−209	759
Foodplain riparian forests	0.97	.32	−1.26	.45	−0.11	.48	5.68	572	−187.5	759.5

*Note*: Positive values of ses.PD, ses.MPD, and ses.MNTD suggest greater diversity and greater phylogenetic distance (superdispersion), while negative values suggest lower diversity and shorter phylogenetic distance (phylogenetic clustering). Values in bold indicate a significant variation.

Abbreviations: Alt, flood height; Ampl, maximum flood amplitude; Plf, maximum depth of the water table.

### 3.3 Species relationships and phylogenetic community structure

The phylogenetic structure analyses revealed that phylogenetic diversity varies along the flooding gradient. A portion of the *terra‐firme* riparian forests exhibited positive ses.PD values, indicating greater phylogenetic diversity and phylogenetic overdispersion within the community. In this portion of the gradient, the ses.MNTD index demonstrated an excess of more recent species (Table 1). In contrast, in all other hydrographic portions, ses.MNTD values were close to zero, suggesting a random phylogenetic structure within each community. The *terra‐firme* riparian zone presented the highest rate of phylogenetic endemism (PE), indicating a greater number of taxa with restricted spatial distribution (Table 1).

**FIGURE 4:**
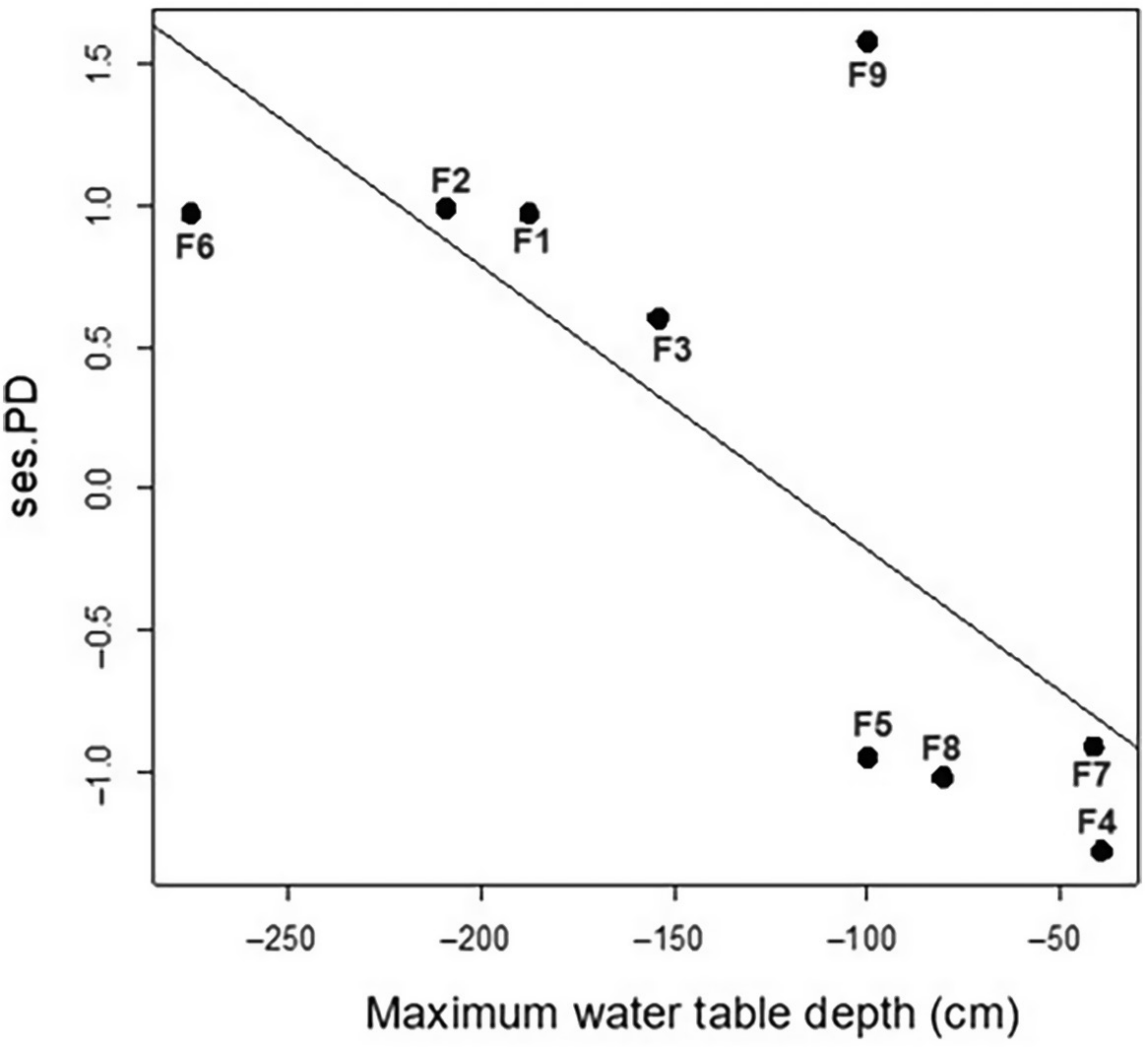
Linear regression between the phylogenetic diversity metrics (ses.PD) and water table depth.

There is a significant reduction in phylogenetic diversity (ses.PD) with increasing water table depth (*p* = .03; *R* ^2^ = 0.52; Figure 4). There is no significant relationship between the flood amplitude and ses.PD (*p* = .22; *R* ^2^ = 0.21), ses.MPD (*p* = .67; *R* ^2^ = 0.03), and ses.MNTD (*p* = .65; *R* ^2^ = 0.03) indices. There is also no significant variation between the standardized values of richness and the maximum depth of the water table (*p* = .69; *R* ^2^ = 0.02), or the maximum height of aboveground flooding (*p* = .15; *R* ^2^ = 0.02) and flood amplitude (*p* = .22; *R* ^2^ = 0.20). No significant relationships were observed between maximum flood height, ses.PD (*p* = .43; *R* ^2^ = 0.09), ses.MPD (*p* = .52; *R* ^2^ = 0.06), and ses.MNTD (*p* = .84; *R* ^2^ = 0.006).

## 4 DISCUSSION

In this study, we demonstrated the significant role of water table depth as a historical determinant in shaping the organization and structure of tree communities across the Amazonian landscape, spanning from *terra‐firme* small streams to *igapó* floodplain riparian forests. By assessing the increasing influence of the flooding pulse, we identified divergent patterns of phylogenetic diversity under varying hydrological conditions (Figure 4). In *terra‐firme* uplands, characterized by shallower water tables and floods mainly caused by local rainfall, riparian forests exhibited higher species richness and phylogenetic diversity (ses.PD, Table 1 and Figure 4), leading to a phylogenetic overdispersion pattern, characterized by more recent relationships between species (Table 1). In contrast, other areas along the hydrographic gradient displayed a random distribution pattern and lower phylogenetic diversity and richness as water table depth increased, thus, supporting the hypothesis, proposed by Wittmann et al. (2010), which suggests that tree lineages were historically filtered by the flooding gradient to occupy similar niches of floodable areas.

The variation in species richness along the flooding gradient reflects complex interactions between flood levels and different plant survival strategies. The establishment of plants in Amazonian wetlands with water deficit or flooding depends on species tolerance and adaptation limits (Maurenza et al., 2009; Parolin, 2001). The phylogenetic approach employed here enabled us to identify ancient dominant lineages in the Amazonian lowlands, including Fabaceae, Lecythidaceae, Bignoniaceae, Sapotaceae, Chrysobalanaceae, and Burseraceae (Hoorn et al., 2010; Lewis, 2005; Lohmann et al., 2013; Luize et al., 2024). The dominant presence of Fabaceae and Lecythidaceae throughout the analyzed gradient underscores their capacities to adapt to diverse Amazonian environments and their fundamental role in sustaining ecosystems (Lavin et al., 2005). Other studies indicate the occurrence of families such as Chrysobalanaceae, Sapotaceae, and Bignoniaceae across a wide diversity of habitats, including different soil types like white sand, and experiencing various flood patterns (Bardon et al., 2013; Gentry, 1982; Jaramillo et al., 2010; Lamarre et al., 2012; Lohmann et al., 2013).

Comparing phylogenetic diversity (ses.PD) and phylogenetic endemism (PE) indices, we found a higher number of restricted lineages in *terra‐firme* riparian forests, which also exhibited greater phylogenetic diversity (Table 1). High PE values were attributed to the presence of restricted families such as Anacardiaceae, Goupiaceae, Nyctaginaceae, and Stemonuraceae, each represented by a single species in this forest. This suggests that taxa adapted to these environment conditions may face challenges in dispersing to other habitats, emphasizing the importance of conserving forests harboring high concentrations of endemic taxa to preserve unique biological diversity and specific ecological niches, ensuring ecological integrity and ecosystem resilience. These results align with those of Rosauer et al. (2009), who observed the highest PE index in an area where a single species with no close relatives was found.

Our results further underscore the role of the water table in regulating tree communities by directly selecting species adapted to soil saturation (Cosme et al., 2017; Costa et al., 2023; Schietti et al., 2014). Accordingly, areas with deeper water tables tend to retain more water, limiting plant establishment and survival for species lacking adaptations to these environmental constraints (Stefanello et al., 2006). This limitation is evident in the reduction in phylogenetic diversity observed in *igapó* riparian forests. Conversely, areas with shallower water table tend to retain less water, reducing or eliminating establishment and survival constraints (Parolin & Wittmann, 2010; Piedade et al., 2013), resulting in increased community phylogenetic diversity, as observed in upland *terra‐firme* riparian forests in this study. This decrease in species richness under more stressful hydro‐edaphic conditions, such as increased flood duration, is consistent with those mentioned in several previous studies (i.e., Assis et al., 2015; Kubitzki, 1989; Moulatlet et al., 2014; Parolin, 2009; Piedade et al., 2013; Prance, 1979; Scudeller & de Souza, 2009). Furthermore, the observed phylogenetic patterns indicate adaptive convergence of different lineages for diversified use of ecological space, influencing community composition and species coexistence (Valencia et al., 1994; de Oliveira and Mori, 1999). Studies demonstrated that floodable forests in the Amazon basin favor species adapted to frequent disturbances (Wittmann et al., 2004; Wittmann & Parolin, 2005), leading to species filtering along the flooding gradient.

The Amazon is considered one of the most important centers of plant diversification in the world (Antonelli et al., 2018). Although some species may have a wide distribution in the Amazon basin, most of them are locally restricted, supporting the environmental complexity of the region (Pitman et al., 2001; ter Steege et al., 2006, 2013). In this study, phylogenetic endemism revealed higher values in *terra‐firme* riparian forests, which also exhibited the greatest phylogenetic diversity (ses.PD). High ses.PD values found in upland riparian forests align with the premise that phylogenetic diversity is greater in communities composed of evolutionarily distinct lineages (Cianciaruso et al., 2009; Magurran, 2004).

These results are consistent with those of studies describing how wetland plant community patterns are mainly influenced by environmental gradients and evolutionary relationships between different tree lineages (Dexter et al., 2017; Fine & Kembel, 2011; Keddy, 1992; Kraft & Ackerly, 2010). The prevalence of several tree families across the Amazon basin during evolutionary time scales reflects, even on a local scale, that independent and distinctive lineages adapted to distinct hydrological gradients, as previously reported for edaphic and topographic gradients (Nelson, 1992; Quesada et al., 2004). Our results support Gentry's (1982) hypothesis regarding habitat specialization driving the diversification of plant groups in the Amazon region. These are also consistent with multidisciplinary studies that state that the current flora of the Amazon is the result of complex ecological, geological, climatic, and hydrological events that acted on landscapes over millions of years (Cracraft et al., 2020; Gopal, 2009; Haffer, 1969; Haffer & Prance, 2002; Hoorn et al., 2010; Kubitzki, 1987; Pennington et al., 2004; Prance, 1985).

*Terra‐firme* forests cover approximately 60% of the Amazon region and harbor significant knowledge gaps regarding its biodiversity (Carvalho et al., 2023; de Oliveira and Mori, 1999; Tuomisto et al., 2003). In this study, the highest phylogenetic diversity and occurrence of families with restricted spatial distribution were observed in riparian forests located along *terra‐firme* low‐order rivers of the drainage system, indicating greater ecosystem stability for this physiognomy (Cadotte et al., 2011). This underscores the urgency of conserving habitats conducive to the colonization of specific ecological niches. Simultaneously, the limited representation of families in regularly flooded forest environments, which comprise about 10% of the Amazon region, highlights the relevance of preserving these unique habitats. Despite limited studies on diversity and phylogenetic structure of plant communities in flooded forests, these areas face significant anthropogenic and climatic pressures (Schöngart et al., 2021).

Considering the anthropogenic climate change's impact on rainfall regimes and flood patterns of Amazon rivers, we anticipate that drastic changes in hydrology may lead to local extinction of habitat specialist tree species and consequently to a significant loss of phylogenetic diversity. Variations in precipitation patterns influence flooding, affecting plant ability to disperse seeds in water. Species adapted to different flooding conditions may gain competitive advantages (Da Silva et al., 2023), thereby shaping the phylogenetic composition of communities over time. River continuum concept (Vannote et al., 1980) emphasizes considering the ecological integrity of the entire rivers and their watersheds for effective management. The spatial arrangement of tree phylogenetic diversity and endemism at local scales emphasizes the need to integrate watersheds into conservation frameworks to protect high phylogenetic diversity and endemism. This study's findings reinforce the critical importance of conserving and researching terrestrial and wetland ecosystems in the Amazon basin, as these areas are home to an invaluable wealth of biodiversity and play a fundamental role in maintaining biodiversity at local, regional, and global scales.

## AUTHOR CONTRIBUTIONS

**Sthefanie do Nascimento Gomes de Souza:** Data curation (equal); formal analysis (equal); investigation (lead); writing – original draft (lead); writing – review and editing (lead). **Darlisson Mesquita Batista:** Data curation (equal); formal analysis (equal); writing – review and editing (equal). **Adriano Costa Quaresma:** Data curation (equal); writing – original draft (equal); writing – review and editing (equal). **Ana Luiza Costa:** Data curation (equal); writing – review and editing (equal). **Layon Oreste Demarchi:** Methodology (supporting); writing – review and editing (equal). **Bianca Weiss Albuquerque:** Methodology (supporting); writing – review and editing (equal). **Viviane Pagnussat Klein:** Methodology (supporting); writing – review and editing (equal). **Gildo Feitoza:** Data curation (equal); writing – review and editing (equal). **Angélica Faria de Resende:** Methodology (supporting); writing – review and editing (equal). **Gisele Biem Mori:** Methodology (supporting); writing – review and editing (equal). **Florian Wittmann:** Writing – original draft (supporting). **Leidiane Leão Oliveira:** Data curation (equal); writing – review and editing (equal). **Amanda Frederico Mortati:** Conceptualization (lead); data curation (equal); writing – review and editing (equal). **Alan Cavalcanti da Cunha:** Data curation (equal); funding acquisition (lead); writing – review and editing (equal). **Jochen Schongart:** Funding acquisition (lead); writing – review and editing (equal). **Aline Lopes:** Writing – original draft (equal); writing – review and editing (equal). **Maria Teresa Fernandez Piedade:** Conceptualization (equal); funding acquisition (lead); supervision (lead); writing – original draft (equal); writing – review and editing (equal). **Thiago André:** Conceptualization (equal); funding acquisition (lead); supervision (equal); writing – original draft (equal); writing – review and editing (equal).

## CONFLICT OF INTEREST STATEMENT

The authors declare that there is no conflict of interest.

## Supporting information


Data S1:

## Data Availability

All relevant data are mentioned in the document and its supporting information files.
