# Correction to “Soil flooding filters evolutionary lineages of tree communities in Amazonian riparian forests”

**DOI:** 10.1002/ece3.70196

**Published:** 2024-08-19

de Souza, S. d. N. G., Batista, D. M., Quaresma, A. C., Costa, A. L., Demarchi, L. O., Albuquerque, B. W., Klein, V. P., Feitoza, G., de Resende, A. F., Mori, G. B., Wittmann, F., Oliveira, L. L., Mortati, A. F., da Cunha, A. C., Schongart, J., Lopes, A., Piedade, M. T. F., & André, T. (2024). Soil flooding filters evolutionary lineages of tree communities in Amazonian riparian forests. *Ecology and Evolution*, 14, e11635. https://doi.org/10.1002/ece3.11635

In the original published version of the article, there was an error in an author's name: Angélica Faria de Resende was incorrectly rendered as Angélica Farias Resende.

The online version of the article has been corrected.

We apologize for this error.

