# Supplementary material for: Soil flooding filters evolutionary lineages of tree communities in Amazonian riparian forests
Source: Ecol Evol. 2024 Jul 23;14(7):e11635. doi: 10.1002/ece3.11635 (PMC11266118; doi:10.1002/ece3.11635)
Supplement: Supplementary file 1 — Data S1: [file ECE3-14-e11635-s001.pdf]

Supplementary material

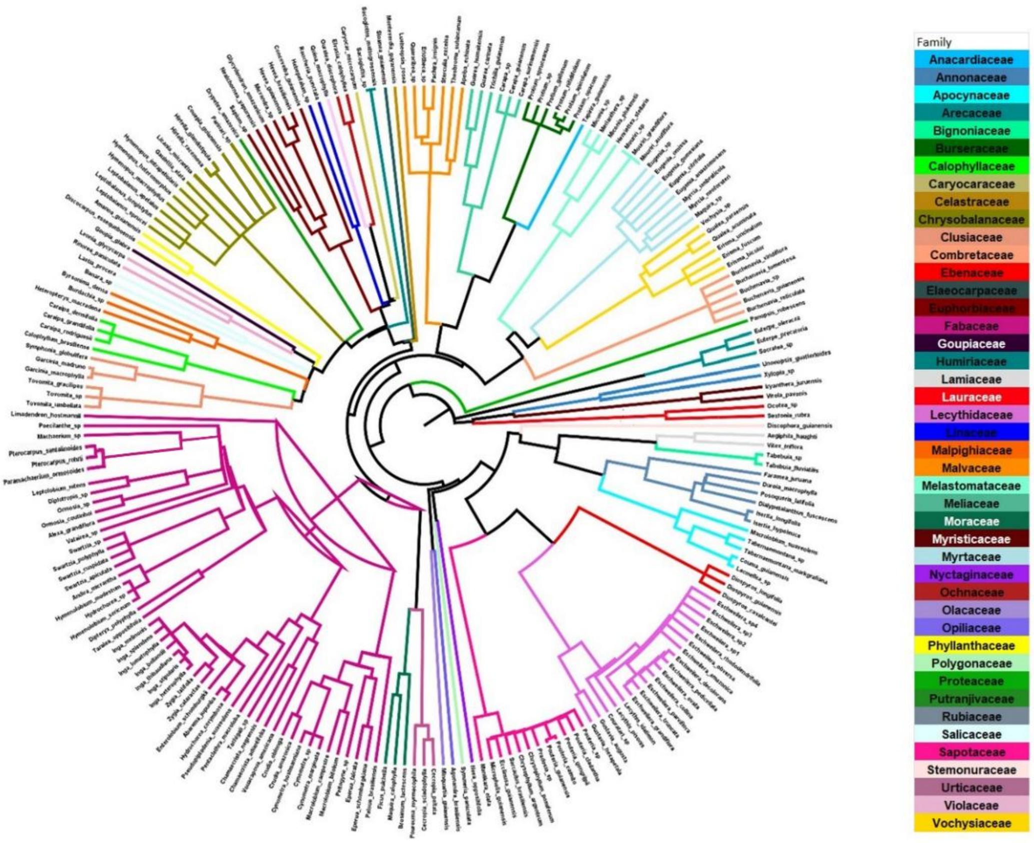

Figure. Phylogenetic tree generated in V.PhyloMaker from the GBOTB megaphylogeny and the list of species from the floristic survey of the Ripária project carried out in the Falsino river plots (eastern Amazon). The different colors represent the identified families.

Table: Matrix of presence and absence of tree species inventoried in the Falsino River along the flood gradient.

**Family/Species**	**Excerpt**		
	**High**	**Medium**	**Low**
**Anacardiaceae**			
Tapirira guianensis Aubl.	x		
**Annonaceae**			
Unonopsis guatterioides (A.DC.) R.E.Fr.		x	x
Xylopia sp.	x	x	x
**Apocynaceae**			
Couma guianensis Aubl.	x		
Lacmellea sp.		x	
Tabernaemontana markgrafiana J.F.Macbr.			x
Tabernaemontana sp.		x	x
**Arecaceae**			
Euterpe oleracea Mart.	x		

Euterpe precatoria Mart.	x		
Socratea sp.	x		
**Bignoniaceae**			
Tabebuia fluviatilis (Aubl.) DC.	x		
Tabebuia sp.	x		
**Burseraceae**			
Protium apiculatum Swart	x		
Protium gallosum Daly	x		
Protium nitidifolium (Cuatrec.) Daly	x		
Protium opacum Swart	x		x
Protium sp.	x		
Protium spruceanum (Benth.) Engl.	x		
**Calophyllaceae**			
Calophyllum brasiliense Cambess.		x	x
Caraipa densifolia Mart.	x	x	x
Caraipa grandifolia Mart.		x	x
Caraipa rodriguesii Paula	x		
**Caryocaraceae**			
Caryocar microcarpum Ducke		x	x
**Celastraceae**			
Monteverdia guyanensis (Klotzsch ex Reissek) Biral		x	x
**Chrysobalanaceae**			
Couepia guianensis Aubl.		x	
Gaulettia elata (Ducke) Sothers & Prance		x	x
Hirtella glandistipula Ducke	x		
Hirtella racemosa Lam.	x		x
Hymenopus heteromorphus (Benth.) Sothers & Prance	x	x	
Hymenopus intrapetiolaris (Spreng. ex Hook.f.)Sothers & Prance	x		
Hymenopus macrophyllus (Benth.) Sothers & Prance		x	x
Leptobalanus apetalus (E.Mey.) Sothers & Prance	x	x	x
Leptobalanus longistylus (Hook.f.) Sothers & Prance			x
Leptobalanus sprucei (Hook.f.) Sothers & Prance	x	x	x
Licania micrantha Miq.	x	x	x
Licania sprucei (Hook.f.) Fritsch			x
Parinari sp.		x	
**Clusiaceae**			
Garcinia macrophylla Mart.	x		
Garcinia madruno (Kunth) Hammel	x	x	x
Symphonia globulifera L.f.	x		
Tovomita gracilipes Planch. & Triana	x		
Tovomita sp.	x		
Tovomita umbellata Benth.	x		
**Combretaceae**			
Buchenavia guianensis (Aubl.) Alwan & Stace	x		
Buchenavia reticulata Eichler		x	x
Buchenavia sp.			x
Buchenavia tomentosa Eichler	x	x	

Buchenavia viridiflora Ducke	x		
**Ebenaceae**			
Diospyros cavalcantei Sothers	x	x	
Diospyros guianensis (Aubl.) Gurke		x	
Diospyros longifolia (Spruce ex Engl.) Sleumer & F.White			x
**Elaeocarpaceae**			
Sloanea guianensis (Aubl.) Benth.	x		x
**Euphorbiaceae**			
Conceveiba guianensis Aubl.	x		
Glycydendron amazonicum Ducke	x		
Hevea brasiliensis (Willd. ex A.Juss.) Mull.Arg.	x		
Hevea guianensis Aubl.			x
Micrandra sp.	x		
Nealchornea yapurensis Huber	x		
Sapium sp.	x		
**Fabaceae**			
Abarema jupunba (Willd.) Britton & Killip			x
Alexa grandiflora Ducke		x	
Andira micrantha Ducke	x	x	
Chamaecrista adiantifolia (Spruce ex Benth.) H.S.Irwin & Barneby	x		
Chamaecrista negrensis (H.S.Irwin) H.S.Irwin & Barneby	x	x	
Crudia amazonica Spruce ex Benth.	x	x	x
Crudia oblonga Benth.			x
Cynometra marginata Benth.		x	
Cynometra sp.		x	
Cynometra hostmanniana Tul.	x	x	x
Diplotropis sp.			x
Dipteryx polyphylla Huber		x	x
Enterolobium schomburgkii (Benth.) Benth.	x	x	x
Eperua schomburgkiana Benth.	x		
Eperua falcata Aubl.	x		
Hydrochorea corymbosa (Rich.) Barneby & J.W.Grimes			x
Hydrochorea sp.		x	
Hymenolobium modestum Ducke		x	
Hymenolobium sericeum Ducke		x	
Inga bollandii Sprague & Sandwith			x
Inga lomatophylla (Benth.) Pittier	x		
Inga splendens Willd.			x
Inga stipularis DC.			x
Inga heterophylla Willd.	x		
Inga melinonis Sagot	x	x	x
Inga thibaudiana DC.		x	
Leptolobium nitens Vogel		x	x
Limadendron hostmannii (Benth.) Meireles & A.M.G.Azevedo	x		
Machaerium sp.	x		x
Macrolobium acaciifolium (Benth.) Benth.		x	
Macrolobium bifolium (Aubl.) Pers.	x	x	x
Macrolobium campestre Huber	x	x	

Macrolobium suaveolens Spruce ex Benth.			x
Ormosia coutinhoi Ducke	x		
Ormosia sp.	x	x	x
Paloue brasiliensis Ducke	x		x
Paramachaerium ormosioides (Ducke) Ducke		x	
Peltogyne sp.	x		
Pentaclethra macroloba (Willd.) Kuntze		x	x
Poecilanthe sp.	x		
Pseudopiptadenia suaveolens (Miq.) J.W.Grimes	x		
Pterocarpus rohrii Vahl	x		x
Pterocarpus santalinoides L'Her. ex DC.	x	x	x
Swartzia apiculata R.S.Cowan			x
Swartzia cuspidata Spruce ex Benth.	x		
Swartzia polyphylla DC.	x		
Swartzia sp.	x		
Tachigali sp.	x		x
Taralea oppositifolia Aubl.	x	x	x
Vatairea sp.			x
Vouacapoua americana Aubl.	x		
Zygia cataractae (Kunth) L.Rico	x	x	x
Zygia latifolia (L.) Fawc. & Rendle			x
**Goupiaceae**			
Goupia glabra Aubl.	x		
**Humiriaceae**			
Sacoglottis mattogrossensis Malme	x		
Sacoglottis sp.		x	
**Lamiaceae**			
Aegiphila haughti Moldenke	x		
Vitex triflora Vahl	x		
**Lauraceae**			
Ocotea sp.	x		
Sextonia rubra (Mez) van der Werff	x		
**Lecythidaceae**			
Couratari sp.	x		x
Eschweilera amazonica R.Knuth	x	x	x
Eschweilera parviflora (Aubl.) Miers	x		
Eschweilera hododendrifolia (R.Knuth) A.C.Sm.			x
Eschweilera collina Eyma	x	x	x
Eschweilera decolorans Sandwith	x	x	x
Eschweilera grandiflora (Aubl.) Sandwith	x		
Eschweilera obversa (O.Berg) Miers		x	x
Eschweilera ovata (Cambess.) Mart. ex Miers	x		
Eschweilera pedicellata (Rich.) S.A.Mori	x	x	x
Eschweilera sp1	x	x	x
Eschweilera sp2	x	x	x
Eschweilera sp3			x
Eschweilera sp4		x	
Eschweilera truncata A.C.Sm.	x		

Gustavia augusta L.		x	x
Gustavia hexapetala (Aubl.) Sm.		x	
Lecythis idatimon Aubl.	x	x	x
Lecythis pisonis Cambess.	x		
**Linaceae**			
Hebepetalum sp.	x		
Roucheria punctata (Ducke) Ducke	x		
**Malpighiaceae**			
Burdachia sp.			x
Byrsonima densa (Poir.) DC.	x	x	
Heteropterys macradena (DC.) W.R.Anderson			x
**Malvaceae**			
Apeiba echinata Gaertn.	x		
Eriotheca sp.	x		
Lueheopsis rosea (Ducke) Burret	x		
Pachira insignis (Sw.) Savigny	x	x	x
Quararibea sp.	x	x	
Sterculia excelsa Mart.	x		
Theobroma subincanum Mart.	x		
**Melastomataceae**			
Henriettea stellaris O.Berg ex Triana			x
Merianthera sp.		x	
Miconia plukenetii Naudin	x		
Miconia sp.			x
Mouriri acutiflora Naudin		x	x
Mouriri grandiflora DC.		x	
Mouriri sp.	x	x	x
**Meliaceae**			
Carapa guianensis Aubl.	x		x
Carapa sp.		x	
Carapa surinamensis Miq.	x		
Guarea carinata Ducke	x		
Guarea humaitensis T.D.Penn.	x		
Trichilia guianensis Klotzsch ex C.DC.			x
**Moraceae**			
Brosimum lactescens (S.Moore) C.C.Berg	x		
Ficus pulchella Schott	x		
Maquira calophylla (Poepp. & Endl.) C.C.Berg		x	x
Maquira sp.		x	
**Myristicaceae**			
Iryanthera juruensis Warb.	x		
Virola pavonis (A.DC.) A.C.Sm.	x	x	x
**Myrtaceae**			
Eugenia anastomosans DC.	x	x	x
Eugenia citrifolia Poir.		x	x
Eugenia gomesiana O.Berg		x	x
Eugenia omissa McVaugh		x	
Eugenia sp.		x	

Myrcia neoforsteri A.R.Lourenco & E.Lucas	x	x	x
Myrcia umbraticola (Kunth) E.Lucas & C.E.Wilson		x	x
**Nyctaginaceae**			
Neea oppositifolia Ruiz & Pav.	x		
**Ochnaceae**			
Elvasia calophyllea DC.			x
Ouratea discophora Ducke			x
Quiina macrophylla Tul.	x		
**Olacaceae**			
Minquartia guianensis Aubl.	x		
**Opiliaceae**			
Agonandra brasiliensis Miers ex Benth. & Hook.f.	x		x
**Phyllanthaceae**			
Amanoa guianensis Aubl.	x		x
Discocarpus essequeboensis Klotzsch	x	x	x
**Polygonaceae**			
Symmeria paniculata Benth.			x
**Proteaceae**			
Panopsis rubescens (Pohl) Rusby		x	x
**Putranjivaceae**			
Drypetes amazonica Steyerm.			x
**Rubiaceae**			
Dialypetalanthus fuscescens Kuhlm.			x
Duroia macrophylla Huber	x		
Faramea juruana K.Krause			x
Isertia hypoleuca Benth.	x		
Isertia longifolia (Hoffmanns. ex Schult.) K.Schum.	x		
Posoqueria latifólia (Rudge) Roem. & Schult.		x	x
**Salicaceae**			
Banara sp.		x	
Laetia procera (Poepp.) Eichler	x		
**Sapotaceae**			
Chrysophyllum argenteum Jacq.	x		x
Chrysophyllum pomiferum (Eyma) T.D.Penn.		x	x
Ecclinusa guianensis Eyma	x		x
Manilkara elata (Allemao ex Miq.) Monach.	x	x	x
Micropholis guyanensis (A.DC.) Pierre	x		
Pouteria caimito (Ruiz & Pav.) Radlk.		x	x
Pouteria cladantha Sandwith	x		
Pouteria gongrijpii Eyma	x		
Pouteria guianensis Aubl.	x		
Pouteria sp.	x	x	x
Pradosia sp.	x		
Sarcaulus brasiliensis Eyma	x		
**Stemonuraceae**			
Discophora guianensis Miers	x		
**Urticaceae**			
Cecropia peltata L.	x		

Cecropia sciadophylla Mart.	x		
Pourouma myrmecophila Ducke	x		
**Violaceae**			
Leonia glycycarpa Ruiz & Pav.	x	x	x
Rinorea paniculata (Mart.) Kuntze			x
**Vochysiaceae**			
Erisma bicolor Ducke	x		
Erisma fuscum Ducke			x
Erisma uncinatum Warm.			x
Qualea acuminata Spruce ex Warm.		x	
Qualea paraensis Ducke	x	x	
Vochysia sp.	x	x	x
